# Congenital Zika syndrome and neuroimaging findings

**DOI:** 10.1590/0100-3984.2017.0206

**Published:** 2017

**Authors:** Beuy Joob, Viroj Wiwanitkit

**Affiliations:** 1 Sanitation 1 Medical Academic Center, Bangkok, Thailand; 2 Visiting Professor, Hainan Medical University, Hainan Sheng, China

Dear Editor,

We read the publication on "Congenital Zika syndrome and neuroimaging findings" with a great interest^(1)^. Ribeiro et al. concluded that "*Although the neuroimaging findings in congenital Zika syndrome are not pathognomonic, many are quite suggestive of the diagnosis, and radiologists should be prepared to interpret such findings accordingly*" and raise a question "what do we know so far?" We would like to share ideas and experience of this topic. In our country, in tropical Southeast Asia, the Zika virus infection is usually asymptomatic^(2)^ and there is usually negative neurological finding. This means that there is not only no pathognomonic finding but also no finding. The use of neuroimaging investigation has limited role in investigation of the case in our setting^(3)^.
