# Supplementary material for: Congenital Zika syndrome and neuroimaging findings
Source: Radiol Bras. 2017 Nov-Dec;50(6):405. doi: 10.1590/0100-3984.2017.0206 (PMC5746886; doi:10.1590/0100-3984.2017.0206)
Supplement: Related texts

# Reply: Congenital Zika syndrome and neuroimaging findings

**Authors:** Bruno Niemeyer de Freitas Ribeiro, Bernardo Carvalho Muniz, Emerson Leandro Gasparetto, Nina Ventura, Edson Marchiori

**Affiliations:** 1 Masters Student, MD, Neuroradiologist at the Instituto Estadual do Cérebro Paulo Niemeyer, Rio de Janeiro, RJ, Brazil; 2 Full Member of the Colégio Brasileiro de Radiologia e Diagnóstico por Imagem (CBR), MD, Neuroradiologist at the Instituto Estadual do Cérebro Paulo Niemeyer, Rio de Janeiro, RJ, Brazil; 3 PhD, MD, Neuroradiologist, Head of the Instituto Estadual do Cérebro Paulo Niemeyer, Rio de Janeiro, RJ, Brazil; 4 PhD, MD, Neuroradiologist at the Instituto Estadual do Cérebro Paulo Niemeyer, Rio de Janeiro, RJ, Brazil; 5 Full Professor at the Universidade Federal do Rio de Janeiro (UFRJ), Rio de Janeiro, RJ, Brazil

Initially, we would like to thank Joob and Wiwanitkit for their letter and their interest in our review article on neuroimaging findings in congenital Zika syndrome (CZS)^(1)^, as well as for affording us the opportunity to discuss the topic further. We believe that this facilitates the exchange of information and the expansion of knowledge about CZS. A careful reading of the letter shows that Joob and Wiwanitkit are doubtful that there are any neuroimaging findings characteristic of CZS, because they deny the existence of this entity among the cases of Zika occurring in their region. However, it is important to mention that our review article was based not on our opinions but on evidence, as well as on an active search of recent articles on the subject published in major international journals, including the New England Journal of Medicine^(2-7)^, Lancet^(8)^, Radiology^(9)^, and others^(10-16)^. Therefore, the data in the current literature are sufficient to foster a belief in the teratogenic potential of the Zika virus^(2,6)^. Nevertheless, we carefully considered whether or not there is a causal relationship between infection with the Zika virus and malformations of the central nervous system, which led to the discussion of the topic in the section "Causality between malformations and congenital infection with the Zika virus" in our article.

Certainly, paradigm shifts require cautious reflection and are sometimes difficult to accept, especially when they are related to a disease that was previously unknown worldwide. The situation is complicated by the fact that the last epidemic of birth defects caused by an infectious pathogen (the rubella virus) occurred more than 50 years ago^(2)^. Ultimately, we believe that personal experiences are valuable and can make important contributions within the scientific community. However, at this moment, we need information based on scientific evidence rather than personal opinions. Further studies should bring greater clarity to this issue.

## References

[1] Ribeiro BNF, Muniz BC, Gasparetto EL (2017). Congenital Zika syndrome and neuroimaging findings what do we
know so far?. Radiol Bras.

[2] Rasmussen SA, Jamieson DJ, Honein MA (2016). Zika virus and birth defects-reviewing the evidence for
causality. N Engl J Med.

[3] Duffy MR, Chen TH, Hancock WT (2009). Zika virus outbreak on Yap Island, Federated States of
Micronesia. N Engl J Med.

[4] Brasil P, Pereira JP Jr, Moreira ME (2016). Zika virus infection in pregnant women in Rio de
Janeiro. N Engl J Med.

[5] Driggers RW, Ho CY, Korhonen EM (2016). Zika virus infection with prolonged maternal viremia and
fetal brain abnormalities. N Engl J Med.

[6] Mlakar J, Korva M, Tul N (2016). Zika virus associated with microcephaly. N Engl J Med.

[7] Hazin AN, Poretti A, Di Cavalcanti Souza Cruz D (2016). Computed tomographic findings in microcephaly associated with
Zika virus. N Engl J Med.

[8] Cauchemez S, Besnard M, Bompard P (2016). Association between Zika virus and microcephaly in French
Polynesia, 2013-15 a retrospective study. Lancet.

[9] Oliveira-Szejnfeld PS, Levine D, Melo ASO (2016). Congenital brain abnormalities and Zika virus what the
radiologist can expect to see prenatally and postnatally. Radiology.

[10] Miner JJ, Cao B, Govero J (2016). Zika virus infection during pregnancy in mice causes
placental damage and fetal demise. Cell.

[11] Noronha L, Zanluca C, Azevedo ML (2016). Zika virus damages the human placental barrier and presents
marked fetal neurotropism. Mem Inst Oswaldo Cruz.

[12] Wang J, Ling F (2016). Zika virus infection and microcephaly: evidence for a causal
link. Int J Environ Res Public Health.

[13] Aragão MFV, van der Linden V, Brainer-Lima AM (2016). Clinical features and neuroimaging (CT and MRI) findings in
presumed Zika virus related congenital infection and microcephaly
retrospective case series study. BMJ.

[14] Werner H, Sodré D, Hygino C (2016). First trimester intrauterine Zika virus infection and brain
pathology prenatal and postnatal neuroimaging findings. Prenat Diagn.

[15] Aragão MFVV, Holanda AC, Brainer-Lima AM (2017). Nonmicrocephalic infants with congenital Zika syndrome
suspected only after neuroimaging evaluation compared with those with
microcephaly at birth and postnatally: how large is the Zika virus
“iceberg”. AJNR Am J Neuroradiol.

[16] Aragão MFVV, Brainer-Lima AM, Holanda AC (2017). Spectrum of spinal cord, spinal root, and brain MRI
abnormalities in congenital Zika syndrome with and without
arthrogryposis. AJNR Am J Neuroradiol.
